# Parkinson's and Alzheimer's diseases in Costa Rica: a feasibility study toward a national screening program

**DOI:** 10.3402/gha.v6i0.23061

**Published:** 2013-12-27

**Authors:** Catharina Wesseling, Norbel Román, Indiana Quirós, Laura Páez, Vilma García, Ana María Mora, Jorge L. Juncos, Kyle N. Steenland

**Affiliations:** 1 Central American Institute for Studies on Toxic Substances (IRET), Universidad Nacional, Heredia, Costa Rica; 2 Institute of Environmental Medicine (IMM), Karolinska Institutet, Stockholm, Sweden; 3 Hospital San Juan de Dios, Social Security of Costa Rica (CCSS), San José, Costa Rica; 4 Clínica de Santo Domingo de Heredia, Social Security of Costa Rica (CCSS), Santo Domingo de Heredia, Costa Rica; 5 Development of Health Services, Social Security of Costa Rica (CCSS), San José, Costa Rica; 6 School of Public Health, University of California at Berkeley, Berkeley, CA, USA; 7 Department of Neurology, Emory University, Atlanta, GA, USA; 8 Rollins School of Public Health, Emory University, Atlanta, GA, USA

**Keywords:** Alzheimer's, Parkinson's, screening, aging, public health system, Costa Rica

## Abstract

**Background:**

The integration of mental and neurologic services in healthcare is a global priority. The universal Social Security of Costa Rica aspires to develop national screening of neurodegenerative disorders among the elderly, as part of the non-communicable disease agenda.

**Objective:**

This study assessed the feasibility of routine screening for Parkinson's disease (PD) and Alzheimer's disease (AD) within the public healthcare system of Costa Rica.

**Design:**

The population (aged ≥65) in the catchment areas of two primary healthcare clinics was targeted for motor and cognitive screening during routine annual health check-ups. The screening followed a tiered three-step approach, with increasing specificity. Step 1 involved a two-symptom questionnaire (tremor-at-rest; balance) and a spiral drawing test for motor assessment, as well as a three-word recall and animal category fluency test for cognitive assessment. Step 2 (for those failing Step 1) was a 10-item version of the Unified Parkinson Disease Rating Scale and the Mini-Mental State Examination. Step 3 (for those failing Step 2) was a comprehensive neurologic exam with definitive diagnosis of PD, AD, mild cognitive impairment (MCI), other disorders, or subjects who were healthy. Screening parameters and disease prevalence were calculated.

**Results:**

Of the 401 screened subjects (80% of target population), 370 (92%), 163 (45%), and 81 (56%) failed in Step 1, Step 2, and Step 3, respectively. Thirty-three, 20, and 35 patients were diagnosed with PD, AD, and MCI, respectively (7 were PD with MCI/AD); 90% were new cases. Step 1 sensitivities of motor and cognitive assessments regarding Step 2 were both 93%, and Step 2 sensitivities regarding definitive diagnosis 100 and 96%, respectively. Specificities for Step 1 motor and cognitive tests were low (23% and 29%, respectively) and for Step 2 tests acceptable (76%, 94%). Based on international data, PD prevalence was 3.7 times higher than expected; AD prevalence was as expected.

**Conclusion:**

Proposed protocol adjustments will increase test specificity and reduce administration time. A routine screening program is feasible within the public healthcare system of Costa Rica.

Mental health and neurological disorders are among the global priorities of the World Health Organization (WHO) (1, 2). Neurodegenerative diseases are incurable conditions that result in progressive degeneration and death of nerve cells leading to problems with motor or mental functioning. Parkinson's disease (PD) and Alzheimer's disease (AD) are major concerns considering the world's aging populations, with the largest increases expected to happen in countries in transition (3). Data on prevalence and incidence are scarce, especially in developing countries, but AD (and other dementias) and PD are listed among the 14 most important causes of disease burden caused by mental illness (3). Mental, neurologic, and substance abuse (MNS) disorders account for an estimated 13% of net global burden of disease, with the greatest weight carried by low- and middle-income countries (LMICs) where these pathologies are widely neglected (1–3). The Disability Adjusted Life-Years (DALYs) for LMICs were recently estimated in 6.8 million for AD and other dementias and 1 million for PD (3).

Despite the public health importance of AD and PD, clinicians in developing countries have insufficient means to adequately diagnose and treat such neurodegenerative disorders. The clinical management of Parkinson's and Alzheimer's patients gets even more complicated with the presence of other chronic diseases common with aging. The physical and cognitive disabilities also affect the quality of life and economic productivity of family members on whom these patients depend for care. Caretakers in developing countries most often lack guidance or professional support. WHO aims at scaling up services for MNS disorders in LMICs (1, 2). This need has also been identified by the Grand Challenges to Global Mental Health (GCGMH) initiative, which includes among its aims to integrate mental health (or MNS disorders) into research, policy, and practice; to integrate care into the non-communicable disease agenda; and to integrate services into priority healthcare platforms (4, 5).

Costa Rica is a medium income developing country in Central America. Life expectancy is high, 79 years in 2011 (6). According to the census of 2011, 7.3% of the 4.5 million population was aged ≥65, and it is estimated that by 2050, this number will have tripled and exceeded the group under age 15 (7). The public health system in Costa Rica has an almost universal coverage. By 2009, 88% of the population was insured by the Social Security of Costa Rica (CCSS) (8). About 80% of the population aged ≥65 uses the services of CCSS (9). Costa Rican health authorities are committed to tackle chronic non-communicable diseases, including neurodegenerative diseases related to aging.

The organizational basis of CCSS consists of approximately 1,000 primary healthcare units with catchment populations of about 4,000, called ‘Basic Teams for Integrated Health Assistance’ (EBAIS), including a physician and a nurse. The CCSS has a routine annual wellness evaluation program for the population aged ≥65 with 65% participation nationwide (Vilma García, CCSS, personal communication). The CCSS anticipates monitoring chronic diseases through this special program, including PD and AD.

This collaborative study aimed to assess the feasibility of a government screening program for neurodegenerative diseases among the elderly in Costa Rica integrated into the universal primary healthcare system, as a platform for improved services, research and evidence-based interventions. The partners were investigators from CCSS and Universidad Nacional in Costa Rica, and Emory University in Atlanta, USA.

## Methods

### Study site and study population

We selected two primary healthcare centers (EBAIS) such that: 1) each would have at least a population of 250 persons aged ≥65; 2) at least 80% of this group would use the CCSS services; and 3) the leading staff would be committed to organizational support. The centers were the urban (formerly rural) EBAIS of Santo Tomás, and the rural EBAIS of Los Ángeles. Both EBAIS refer to the Clinic of Santo Domingo de Heredia and are close to the capital San José. The goal was to screen 500 patients. Subjects were recruited between January 2010 and January 2011.

### Ethical considerations

The ethical committees of the Universidad Nacional, CCSS, and Emory University approved the study. After the attending nurse explained the study, verbal consent was obtained since the screening was conducted as part of the routine annual health check-up and comprised only non-invasive methods. There were no refusals.

### Screening methods

**Fig. 1:**
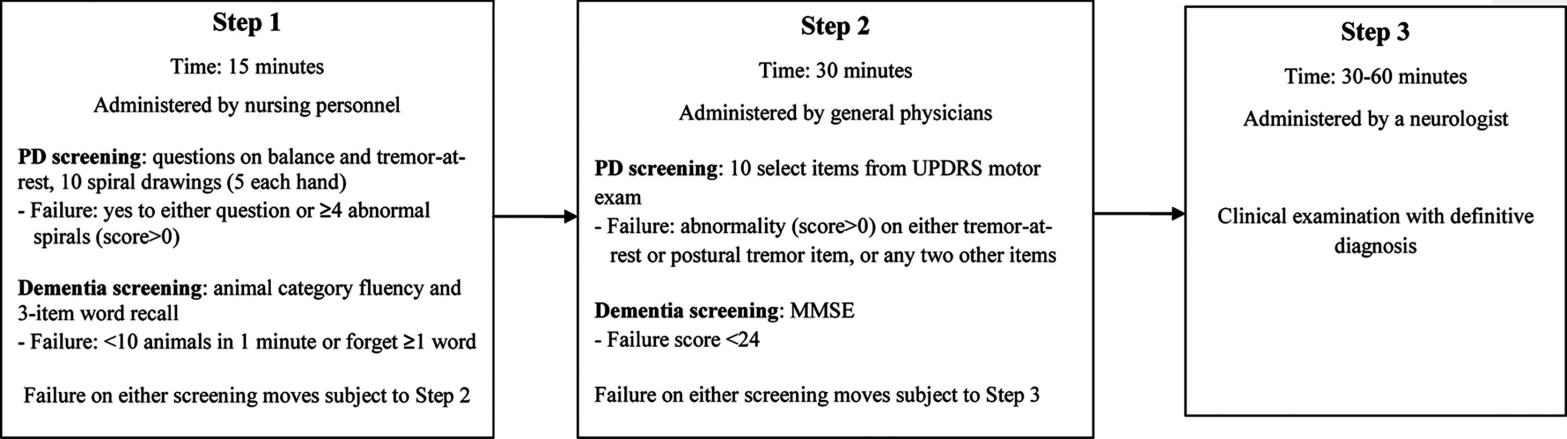
Design of screening protocol for neurodegenerative diseases among the elderly in Costa Rica.

The screening protocol involved a tiered three-step approach, using tests of proven validity. Each tier had progressively higher specificity. Since the application of the protocol added to the routine work of the staff, time was a prime consideration. The protocol is outlined in Fig. 1.

### Step 1

Step 1 screening was administered to all participants and designed to take approximately 15 min. It included a 5-min questionnaire, two motor-symptom questions, one motor test, and two cognitive tests. The questionnaire inquired demographic data (age, sex, most common occupation, education, and district of residence) and contained a few questions to explore an association between pesticides and Parkinson disease (10), specifically smoking status, history of work in agriculture and, if positive, years of occupational exposure to pesticides.

The ‘yes/no’ motor-symptom questions addressed tremor-at-rest and balance: 1) ‘During the last year, have you had tremors in one or both hands when at rest?’ and 2) ‘During the last year, has it become difficult for you to maintain your balance when walking?’. The motor test was a spiral drawing test commonly used in the assessment of Parkinsonian motor signs (11–13), consisting of drawing 10 spirals with each hand on paper sheets placed on top of a digitizing tablet, the results of which were fed into a laptop computer. Due to computer unfamiliarity, both nurses and patients perceived the computerized spiral tests as stressful. Therefore, after screening about 20% of the targeted patients (*n*=107), the protocol was changed to manual drawing of five spirals with each hand to reduce administration time to less than 5 min. Spirals were scored by the nurses as normal (score 0) and abnormal (score 1–4), based on the familiarity with multiple examples of normal spirals.

The two cognitive tests took only a few minutes: a word-recall test after distraction (the patient was asked to remember the words ‘house, shoe, paper’, and then to repeat them after answering the symptom questions), and a category fluency test naming as many animals as possible in 1 min.

Screening positive in Step 1 for potential PD was defined as answering positively to any of the two-symptom questions OR scoring ≥1 on at least four spirals in either hand. Screening positive in Step 1 for potential AD was defined as failing ≥1 word in the word-recall test OR naming <10 animals in 1 min.

### Step 2

Step 2 was administered to those failing Step 1 by general practitioners working at the two EBAIS, the majority by a single physician (IQ). Step 2 was designed to distinguish between less specific motor and cognitive indicators and more specific early signs of PD and AD. The tests were an abbreviated version of the Unified Parkinson Disease Rating Scale (UPDRS) examination and the Mini-Mental State Examination (MMSE) questionnaire, validated scales commonly used in the screening of motor and cognitive signs, respectively.

The UPDRS version we used was a modified form of the 10-item motor subscale proposed by Louis et al. (14). Tremor-at-rest was hypothesized *a priori* as the most sensitive item for detection of PD. Other items in the scale include speech (hypophonia), facial expressivity, axial and appendicular rigidity, bradykinesia, and abnormalities of posture and gait. In an attempt to also capture atypical presentations of PD (estimated at 20%), we also included items of postural tremor of the hands and finger tapping as a test of fine motor dexterity. By including postural tremor, we avoided excluding PD subjects who may present with essential (typically postural) tremor that later develops to PD, or patients with Lewy Body disease presenting with motor symptoms before developing dementia. Finger tapping is part of the original UPDRS and it was meant to capture characteristic abnormalities of fine motor dexterity in PD in patients without tremor (i.e. akinetic-rigid subtype). All items were scored from 0 to 2. A score of 1 indicated mild abnormality, and a score of 2 abnormality of moderate or greater severity. The application of this abbreviated UPDRS version took approximately 10 min.

The MMSE is a 10-item questionnaire that evaluates cognitive domains of orientation, registration, concentration, memory, language, and praxis. The scores range between 0 and 30 points, and the cutoff for abnormality is <24. It has been validated in Spanish (15), and it is widely used in Latin America in clinical practice (16, 17), including in the CCSS. The administration of the MMSE took about 15 min but longer in patients with cognitive deficits.

Screening positive in Step 2 for potential PD was defined as any abnormality (score >0) on the tremor-at-rest or postural tremor items of the UPDRS, OR a score >0 on at least two other items. Screening positive in Step 2 for potential AD was defined as a MMSE score <24, without adjustment for age, gender, or education.

### Step 3

Step 3 of the screening protocol involved a clinical evaluation of those failing Step 2 by a neurologist specialized in aging disorders (NR). The diagnostic criteria for PD were based on the UK Brain Bank criteria (18), defined as bradykinesia plus tremor-at-rest and/or cogwheel rigidity. For AD and mild cognitive impairment (MCI), diagnoses were made in agreement with current guidelines (19–21). The neurologist classified subjects as either possible/probable PD (possible PD patients presented with typical Parkinson's features including bradykinesia but without tremor), possible/probable AD, or MCI. Subjects who did not fall in these categories were considered normal or were diagnosed with other neurologic, rheumatologic, or orthopedic problems common in the elderly. The Step 3 motor examination took about 15 min and the cognitive examination about 30 min.

### Training, communication, and feedback

The nursing personnel were thoroughly trained in the administration of the questionnaire and cognitive tests of Step 1. The neurologists (JJ and NR) trained the nurses and the general practitioners in the administration of the Step 1 spiral drawing test and the Step 2 UPDRS and MMSE tests, respectively. A trained field manager monitored the nurses in charge of Step 1 screening. Consistency in Step 2 assessments was reinforced and monitored by one neurologist (NR). During data collection, meetings and workshops were organized with the leading staff and those who conducted the screening, to evaluate progress and make adjustments as needed. At the end, an open-ended evaluation questionnaire was applied to all collaborating health personnel.

### Statistical analyses

Analyses consisted of descriptive statistics of the demographics of the study subjects and test results. Sensitivity, specificity, and positive predictive values (PPV) were calculated for specific screening tests and for the overall failure criteria in relation to the next step as established in the protocol, assuming that those who had tested negative in the previous step were healthy.

The prevalence of PD, AD, and MCI in the study population was calculated. The expected number of PD cases in our study population was nine, derived from age-specific prevalence (0.5% ages 60–69, 1.5% ages 70–79, 3% ages ≥80) based on an international review of nine population-based studies in Europe and Asia (22). The expected number for AD cases in our study population was 21, derived from age-specific prevalence (2% ages 70–79, 20% ages ≥80) based on US estimates (23). For MCI no expected numbers could be calculated, due to scarcity of reliable population prevalence data. The numbers of observed PD and AD cases were compared to the expected numbers.

## Results

The 401 screened subjects represented about 80% of the population aged ≥65 living in the catchment areas of the two EBAIS. A few individuals aged 60–64 were also inadvertently screened (2%). Table 1 describes the study population. More women than men were tested (60 vs. 40%), almost a third was aged <70, 72% had primary education or less, and about 60% had worked in agriculture.

**Table 1:** Demographic characteristics of the study participants

	Men, *N*=159 *n* (%)	Women, *N*=242 *n* (%)	Total, *N*=401 *n* (%)
Health clinic			
Santo Tomás	95 (59.8)	151 (62.4)	246 (61.4)
Los Ángeles	64 (40.2)	91 (37.6)	155 (38.6)
Age, years			
60–69*	44 (27.7)	86 (35.5)	130 (32.4)
70–74	54 (34.0)	58 (24.0)	112 (27.9)
75–79	19 (11.9)	54 (22.3)	73 (18.2)
≥80	42 (26.4)	44 (18.2)	86 (21.5)
Agricultural worker	117 (73.6)	119 (49.2)	236 (59.0)
Pesticide use	63 (39.6)	10 (4.1)	73 (18.2)

* Eight subjects were aged between 61 and 64 years, all others were aged between 65 and 69.

Table 2 summarizes the flow of screened subjects from Step 1 to the definitive diagnosis in Step 3. In Step 1, 370 (92%) subjects failed: 329 (82%) failed motor testing (primarily the spiral drawing test) and 297 (74%) failed cognitive testing (primarily the three-word-recall test), with 256 (64%) failing both motor and cognitive testing; only 31 (8%) of the patients passed all of the tests.

**Table 2:** Patient flow in a three-step screening procedure for Parkinson's disease (PD), Alzheimer's disease (AD), or mild cognitive impairment (MCI)

Step	Subjects screened, *n* a	Motor failure only, *n* (%)	Cognitive failure only, *n* (%)	Motor and cognitive failure, *n* (%)	Total abnormal, *n* (%)	Normal, *n* (%)
1	401	73 (18)	41 (10)	256 (64)	370 (92)	31 (8)
2	364	79 (22)	33 (9)	51 (14)	163 (45)	201 (55)
3	145	(PD) 26 (18)	(MCI/AD) 48 (33)	(PD+MCI/AD) 7 (5)	81 (56)	64 (44)^b^

a Step 2 and Step 3 include, respectively, 6 and 3 subjects who tested negative in the previous steps.

b 24 patients diagnosed as normal and 40 with a diagnosis other than PD, AD, or MCI.

In Step 2, further screening tests were applied to 364 patients and, of these, 163 (45%) failed: 130 (36%) failed the UPDRS motor evaluation and 84 (23%) the MMSE cognitive evaluation, with 51 (14%) failing both motor and cognitive tests; 201 (55%) subjects passed both tests.

In Step 3, 145 of the 163 subjects who failed Step 2 underwent a neurologic examination. Of the 21 who were not examined, four had died and 17 could not be contacted or were unable to attend. Eighty-one (56%) subjects were diagnosed with a neurodegenerative disease: 33 with possible/probable PD, 20 with possible/probable AD, and 35 with MCI, with seven of these cases having a combined diagnosis of PD with MCI or AD. Of the remaining patients, 24 did not have a specific disorder and 40 had other diagnoses that explained motor or cognitive failure in Step 2, including psychiatric and mood disorders, disorders of the peripheral nervous system, and musculoskeletal disorders. Tremor-related pathologies were present in 34 of these patients (19 essential, 11 drug-induced and four cerebellar tremors).

Step 1 overall motor and cognitive failure criteria had a high sensitivity to predict failure on the UPDRS and MMSE of Step 2 (both 93%) but a low specificity (23 and 29%, respectively) (Table 3). The results for the motor subtests of Step 1 varied, with the self-reported symptom of tremor-at-rest having a higher PPV than the balance symptom and spiral drawing test. Regarding the cognitive subtests, the animal fluency test was less sensitive but more specific and had a higher PPV than the word-recall test. The sensitivity and specificity of Step 2 UPDRS for PD were, respectively, 100 and 76%, and of MMSE for AD/MCI were 96 and 94%.

**Table 3:** Sensitivity, specificity, and positive predictive value (PPV) for tests used in the screening for Parkinson's disease (PD) and for Alzheimer's disease (AD) or mild cognitive impairment (MCI)^a^

Step 1 tests versus Step 2 tests				Step 2 tests versus Step 3 diagnoses			
	
	Sensitivity	Specificity	PPV		Sensitivity	Specificity	PPV
			
	Step 2 motor failure (UPDRS)				Step 3 PD diagnosis		
Step 1 overall motor failure criteria (*n*=388)^b^	93.1	22.5	37.7	Step 2 motor failure (UPDRS) (*n*=367)^d^	100	75.8	28.3
Symptoms, any of 2 positive (*n*=385)	62.0	71.1	51.9				
Tremor (*n*=386)	40.3	90.3	67.5				
Balance (*n*=387)	46.9	75.5	49.2				
Spiral drawing failure (*n*=382)	90.5	28.5	38.4				
	Step 2 cognitive failure (MMSE)				Step 3 MCI or AD diagnosis		
			
Step 1 overall cognitive failure criteria (*n*=380)^c^	92.8	29.0	26.7	Step 2 cognitive failure (MMSE) (*n*=367)^e^	96.4	93.6	72.6
Animal fluency <10 (*n*=388)	51.2	82.6	52.3				
Word recall <3 (*n*=378)	88.0	32.5	26.8				

a The denominators in Step 2 include 24 subjects with negative motor and cognitive test results in Step 1, assumed to be disease-free and untested in Step 2; the denominators in Step 3 include 199 subjects with negative motor and cognitive test results in Step 2, assumed to be disease-free and untested in Step 3.

b Overall motor failure criteria, Step 1: any of the two-symptom questions positive OR ≥ 4 abnormal spiral drawings on either hand.

c Overall cognitive failure criteria, Step 1: animal fluency <10 OR word recall<3.

d Step 2 motor failure: abnormal tremor-at-rest OR abnormal postural tremor OR ≥ 2 other abnormal UPDRS items.

e Step 2 cognitive failure: MMSE score<24.

Only four of the 33 patients with possible/probable PD had a previous diagnosis of PD, and two of the 35 MCI patients and two of the 20 AD patients had a previous diagnosis of memory problems. Overall, 90% of the cases detected in this screening pilot were new. The prevalence in the study population (with untested patients in Step 2 and Step 3 in the denominator) was 8% for PD, 5% for AD, and 9% for MCI, with no differences between men and women. The observed number of PD cases (*n*=33) was 3.7 times higher than the nine expected cases. The number of observed AD cases (*n=*20) was similar to the expected number of 21.

Annex 1 contains detailed results for the screening parameters of all tests of cognition and motor control used in this study in relation to the final diagnosis, according to different cutoff points and failure criteria.

In the final qualitative evaluation, all health personnel valued an early detection system for neurodegenerative diseases as important and feasible. The extra workload was a concern, which sometimes affected motivation, in particular among the nurses. Due to the low specificity of the Step 1 tests, the workload was much higher than anticipated for the general physicians participating in Step 2. The customary time allocated per patient was insufficient to administer the MMSE and UPDRS in addition to other routine medical tests. In addition, the only on-site neurologist participating in the project (NR) had to make a considerable effort to examine the 145 patients who passed to Step 3, a much higher number than expected. Nonetheless, these obstacles can be addressed with improvements in the screening protocol (see discussion) and the central health authorities in San José are keen to extend this pilot experience.

## Discussion

This study assessed the feasibility of an early detection system of neurodegenerative disease among the elderly in the Costa Rican public health system. Development of such a system would have significant public health implications and is in line with the goals of WHO and the GCGMH (1–5).

The tiered three-step approach allowed evaluation of a number of simple tests for a future governmental screening program in this field. We used broad criteria for failure in Step 1 to try to avoid false negatives and to be able to reasonably estimate the prevalence of neurodegenerative diseases in this population during Step 3. The overall motor and cognitive failure criteria at this first screening level lacked specificity (respectively 23% and 29% only), which resulted in too many patients passing on to Step 2 to be manageable on a larger scale.

The main problem in Step 2 was the time required for even the abbreviated UPDRS test and the MMSE test. We needed to double the allotted time to examine the elderly. Although the sensitivities and specificities of the UPDRS and MMSE were acceptable to good, the large number of patients examined in Step 2 and the low PPV of the UPDRS caused more patients than expected to pass on to Step 3. This passage rate would overwhelm the neurologist's service in a large-scale endeavor.

Considering these results, we examined different cutoff points that would increase the specificity without lowering the sensitivity of Step 1 tests too much, and assessed the possibility of simplification of the Step 2 tests to shorten administration time. With regard to the motor component of Step 1, although changes in cutoff points for subtests and overall failure criteria considerably improved the specificity (data not shown), a more effective solution is to eliminate Step 1 motor tests altogether and, instead, administer the UPDRS as Step 1 and Step 2. Moreover, our results show that when using just the two tremor items (tremor-at-rest and postural tremor), there was only a minimal loss of sensitivity from 100 to 97%, whereas the specificity slightly increased from 76 to 78% (Table 4). It seems feasible to train nurses and general physicians in the routine administration of these rather simple test items to all elderly patients attending the CCSS health centers. However, the PPV will still be only 30%, implying that a large number of false positives will be referred for neurological evaluation. The development of an intermediate screening test for tremor-positive patients may therefore be desirable. However, from a public health perspective, based on the results of this study, most of the ‘false positives’ in Step 3 would have other significant and treatable pathologies, in many cases not previously diagnosed.

**Table 4:** Recommended changes in screening protocol, with parameters of sensitivity, specificity, and positive predictive value (PPV) for new failure criteria in each step, and with an estimated improvement in screening efficiency as compared to the current protocol

New failure criteria		Sensitivity	Specificity	PPV	Reduction in subjects in next step	Cases that would have been missed
**Motor screening**						
Step 1–2	Eliminate current Step 1	n.a.	n.a.	n.a.	−401 (100%)	0
	Abnormal results in any of two tremor items of UPDRS	96.9^a^	78.2^a^	29.8^a^	−9 (5.5%)^a^	1/33 PD (3%)
**Cognitive screening**						
Step 1	Word recall <2 words OR Animal fluency <10	78.3^b^	59.0^b^	36.7^b^	−111 (37%)^b^	5/35 MCI (14%) 1/20 AD (5%)
Step 2	No changes (MMSE <24)	96.4^c^	93.6^c^	72.6^c^	0^c^	0

a The screening parameters for the new recommended Step 1 and Step 2 failure criterion relate to Step 3 PD diagnosis.

b The screening parameters of the new recommended overall failure criteria for Step 1 relate to MMSE of Step 2.

c The screening parameters are the same as in the original protocol and relate to Step 3 MCI/AD diagnosis.

With regard to the cognitive component of Step 1, allowing the patients to forget one word in the recall test (as opposed to none in the current study) and maintaining the category fluency test unchanged, the specificity of the overall cognitive failure criteria of Step 1 increased from 29 to 59%, preventing more than 100 false positives from continuing to Step 2 as compared to the current criteria (Table 4). On the downside, 19 abnormal MMSE would be missed, with five MCI (14%) and one AD (5%) cases among them. For Step 2, the sensitivity and specificity of MMSE are good and few false positives continued to Step 3. Although age and education significantly predicted the MMSE score in our data, little was gained in prediction by setting a lower failure cutoff point (score <23) for patients over age 80 or with low literacy.

Not all patients who entered Step 1 had a Step 2 test or a final Step 3 diagnosis, limiting our ability to estimate true sensitivity and specificity for one step regarding the next. This is inevitable in a real-world situation where time is of the essence and medical staff cannot evaluate in a later step those who were negative in a previous one. In calculating sensitivity and specificity, we have included in the denominator of Step 2 and Step 3 the patients who had scored negatively in Step 1 and Step 2 (24 and 199, respectively), under the assumption that they were healthy. Although reasonable, this implies some inevitable misclassification of impaired persons as healthy.

With the high sensitivities for Step 1 and Step 2 failures and the likely low number of missed cases, the project allowed the estimation of the prevalence of neurodegenerative disorders in the study population, which was considerably higher than expected for PD and about as expected for AD. The number of PD cases detected was almost double the cases of AD, although AD is a more common disease than PD (24). Likewise, essential tremor was less prevalent than PD in the examined population. Prevalence depends on the quality of the diagnosis, but the diagnoses were made on the basis of established criteria. It is possible that AD may have been more present in the 20% of the target population that never came to the health center, or among the 33 patients lost to follow-up in Step 2 and Step 3. The association in our study between pesticide exposure and PD (10) could partially explain our findings of a high prevalence of PD.

The CCSS aspires to address chronic neurodegenerative diseases through a national screening program linked to an existing priority program on non-communicable diseases within the primary healthcare system, as advocated by WHO and the GCGMH initiative (1–5). Such a system will be linked to interventions and research. CCSS, in fact, is dedicating considerable resources to reach these goals. The patients diagnosed with a neurodegenerative disease in this study are now part of an intervention program. Furthermore, since this project was conducted, two national dementia associations have been established and Costa Rican clinicians at CCSS have joined the 10/66 Dementia Research Group of Alzheimer's Disease International (http://www.alz.co.uk/1066/research.php), the latter not only addressing patients’ needs but also that of caretakers. We conclude that a routine screening program in the public health system of Costa Rica is feasible and can lead to improved care, research, and preventive actions. To our best knowledge, such a screening program would be the first worldwide.

## Appendix

**Annex 1:** Screening parameters according to different failure criteria and cutoffs for tests of cognition and motor control used in Step 1 and Step 2 of the screening protocol in relation to final diagnosis of Alzheimer's disease (AD), mild cognitive impairment (MCI), or Parkinson's disease (PD): sensitivity (sens), specificity (spec), positive predictive value (PPV), negative predictive value (NPV), false positive rate (FPRate), and false negative rate (FNRate)

Tests for cognitive evaluation									
**Word-recall test**									
		Clinical diagnosis of AD or MCI by a neurologist							
		
Cutoffs for test failure	*N*= 359	Pos	Neg	Sens	Spec	PPV	NPV	FPRate	FNRate
0 word recall	Pos	27	27	52.9	91.2	50.0	92.1	8.8	47.1
	Neg	24	281						
1 word recall	Pos	39	86	72.2	71.8	31.2	93.6	28.2	27.8
	Neg	15	219						
2 word recall*	Pos	52	205	96.3	32.8	20.2	98.0	67.2	3.7
	Neg	2	100						
**Animal fluency test**									
		Clinical diagnosis of AD or MCI by a neurologist							
		
Cutoffs for test failure	*N*=368	Pos	Neg	Sens	Spec	PPV	NPV	FPRate	FNRate
<6 animals	Pos	10	7	18.2	97.8	58.8	87.2	2.2	81.8
	Neg	45	306						
<7 animals	Pos	14	11	25.5	96.5	56.0	88.0	3.5	74.5
	Neg	41	302						
<8 animals	Pos	19	20	34.5	93.6	48.7	89.1	6.4	65.5
	Neg	36	293						
<9 animals	Pos	26	33	47.3	89.5	44.1	90.6	10.5	52.7
	Neg	29	280						
<10 animals*	Pos	33	55	60.0	82.4	37.5	92.1	17.6	40.0
	Neg	22	258						
<11 animals	Pos	37	82	67.3	73.8	31.1	92.8	26.2	32.7
	Neg	18	231						
<12 animals	Pos	44	121	80.0	61.3	26.7	94.6	38.7	20.0
	Neg	11	192						
<13 animals	Pos	47	170	85.5	45.7	21.7	94.7	54.3	14.5
	Neg	8	143						
<14 animals	Pos	50	205	90.9	34.5	19.6	95.6	65.5	9.1
	Neg	5	108						
<15 animals	Pos	53	234	96.4	25.2	18.5	97.5	74.8	3.6
	Neg	2	79						
**Mini-Mental State Examination (MMSE)**									
		Clinical diagnosis of AD or MCI by a neurologist							
		
Cutoffs for test failure	*N*=343	Pos	Neg	Sens	Spec	PPV	NPV	FPRate	FNRate
MMSE score<18	Pos	14	1	25.5	99.7	93.3	87.5	0.3	74.5
	Neg	41	287						
MMSE score<19	Pos	20	2	36.4	99.3	90.9	89.1	0.7	63.6
	Neg	35	286						
MMSE score<20	Pos	25	2	45.5	99.3	92.6	90.5	0.7	54.5
	Neg	30	286						
MMSE score<21	Pos	27	6	49.1	97.9	81.8	91.0	2.1	50.9
	Neg	28	282						
MMSE score<22	Pos	33	6	60.0	97.9	84.6	92.8	2.1	40.0
	Neg	22	282						
MMSE score<23	Pos	37	13	67.3	95.5	74.0	93.9	4.5	32.7
	Neg	18	275						
MMSE score<24*	Pos	53	20	96.4	93.1	72.6	99.3	6.9	3.6
	Neg	2	268						
MMSE score<25	Pos	53	35	96.4	87.8	60.2	99.2	12.2	3.6
	Neg	2	253						
MMSE score<26	Pos	53	61	96.4	78.8	46.5	99.1	21.2	3.6
	Neg	2	227						
MMSE score<27	Pos	55	100	100.0	65.3	35.5	100.0	34.7	0.0
	Neg	0	188						

**Tests for motor evaluation**									

**Symptom questions**									
		Clinical diagnosis of PD by a neurologist							
		
Failure criteria	*N*=365–368	Pos	Neg	Sens	Spec	PPV	NPV	FPRate	FNRate
Tremor	Pos	15	58	45.5	82.6	20.5	93.9	17.4	54.5
	Neg	18	275						
Balance symptoms	Pos	14	100	42.4	70.1	12.3	92.5	29.9	57.6
	Neg	19	234						
Tremor and balance	Pos	7	36	21.2	89.3	16.3	92.0	10.7	78.8
	Neg	26	299						
Tremor or balance*	Pos	22	122	66.7	63.3	15.3	95.0	36.7	33.3
	Neg	11	210						
**Spiral drawing test**									
		Clinical diagnosis of PD by a neurologist							
		
Cutoffs for test failure	*N*=363	Pos	Neg	Sens	Spec	PPV	NPV	FPRate	FNRate
≥1 abnormal spiral	Pos	32	290	97.0	12.1	9.9	97.6	87.9	3.0
	Neg	1	40						
≥2 abnormal spirals	Pos	31	278	93.9	15.8	10.0	96.3	84.2	6.1
	Neg	2	52						
≥3 abnormal spirals	Pos	31	265	93.9	19.7	10.5	97.0	80.3	6.1
	Neg	2	65						
≥4 abnormal spirals*	Pos	30	250	90.9	24.2	10.7	96.4	75.8	9.1
	Neg	3	80						
≥5 abnormal spirals	Pos	29	236	87.9	28.5	10.9	95.9	71.5	12.1
	Neg	4	94						
≥6 abnormal spirals	Pos	28	214	84.8	35.2	11.6	95.9	64.8	15.2
	Neg	5	116						
≥7 abnormal spirals	Pos	28	178	84.8	46.1	13.6	96.8	53.9	15.2
	Neg	5	152						
≥8 abnormal spirals	Pos	26	150	78.8	54.5	14.8	96.3	45.5	21.2
	Neg	7	180						
≥9 abnormal spirals	Pos	22	127	66.7	61.5	14.8	94.9	38.5	33.3
	Neg	11	203						
≥10 abnormal spirals	Pos	22	103	66.7	68.8	17.6	95.4	31.2	33.3
	Neg	11	227						
**Unified Parkinson Disease Rating Scale (UPDRS) abbreviated version**									
		Clinical diagnosis of PD by a neurologist							
		
Failure criteria for abnormal UPDRS items	*N*=341–351	Pos	Neg	Sens	Spec	PPV	NPV	FPRate	FNRate
Speech (hypophonia)	Pos	0	7	0.0	97.7	0.0	90.5	2.3	100.0
	Neg	32	304						
Abnormal facial expressivity	Pos	4	12	12.5	96.1	25.0	91.4	3.9	87.5
	Neg	28	299						
Tremor-at-rest	Pos	29	54	90.6	82.6	34.9	98.8	17.4	9.4
	Neg	3	257						
Postural tremor	Pos	27	58	84.4	81.4	31.8	98.1	18.6	15.6
	Neg	5	253						
Neck rigidity	Pos	5	5	15.6	98.4	50.0	91.8	1.6	84.4
	Neg	27	304						
Arm rigidity	Pos	7	8	21.9	97.4	46.7	92.3	2.6	78.1
	Neg	25	300						
Finger tapping	Pos	22	17	68.8	94.5	56.4	96.7	5.5	31.3
	Neg	10	294						
Stooped posture	Pos	11	29	34.4	90.6	27.5	93.0	9.4	65.6
	Neg	21	281						
Parkinsonian gait	Pos	10	47	31.3	85.3	17.5	92.5	14.7	68.8
	Neg	22	272						
Axial bradykinesia	Pos	3	5	9.7	98.4	37.5	91.6	1.6	90.3
	Neg	28	305						
Tremor-at-rest or postural tremor	Pos	31	73	96.9	76.5	29.8	99.6	23.5	3.1
	Neg	1	238						
Tremor-at-rest and postural tremor	Pos	25	39	78.1	87.5	39.1	97.5	12.5	21.9
	Neg	7	272						
Any tremor or at least two other items*	Pos	32	81	100.0	74.0	28.3	100.0	26.0	0.0
	Neg	0	230						
Any tremor or finger tap	Pos	31	75	96.9	75.9	29.2	99.6	24.1	3.1
	Neg	1	236						
Any tremor or abnormal gait	Pos	32	95	100.0	73.3	25.2	100.0	26.7	0.0
	Neg	0	261						

* The criteria for failure used in the current screening pilot study.

Note: These analyses include all patients who were examined in Step 1, Step 2, and Step 3 (*n*=145), or were examined in Step 1 and Step 2 and considered healthy in Step 2 (*n*=199), or were only examined in Step 1 and were considered healthy (*n*=24). Not included were the 33 patients who were lost to follow-up after Step 1 (*n*=12) or after Step 2 (*n*=21). Different numbers are due to missing data for specific tests or test items.
